# Case Report: Euglycemic ketoacidosis in a non-diabetic patient: a rare adverse effect of SGLT2 inhibitor therapy

**DOI:** 10.3389/fendo.2025.1746210

**Published:** 2026-01-16

**Authors:** Sai Prasad, Shreya Sharma, Rudrakshi Shetty, Snigdha Singh, Vidhi Parmar, Channabasappa Shivaprasad, Vidhi Dhaduk

**Affiliations:** 1 Department of Internal Medicine, S Nijalingappa Medical College Navanagar, Bagalkote, Karnataka, India; 2 Department of Internal Medicine, Hamdard Institute of Medical Sciences and Research, New Delhi, Delhi, India; 3 Nanavati Max Super Speciality Hospital, Mumbai, Maharashtra,, India; 4 Department of Pediatrics, University of New Mexico Children’s Hospital, Albuquerque, NM, United States; 5 D Y Patil Medical College, Navi Mumbai, Maharashtra, India; 6 Department of Endocrinology, Sapthagiri Institute of Medical Sciences, Bengaluru, Karnataka, India; 7 Department of Internal Medicine, Smt. Shantabaa Medical College and General Hospital Amreli, Ahmedabad, India

**Keywords:** dapagliflozin, euglycemic ketoacidosis, heart failure, nondiabetic, SGLT2 inhibitor

## Abstract

**Background:**

Sodium-glucose cotransporter-2 (SGLT2) inhibitors are increasingly prescribed for heart failure and chronic kidney disease, irrespective of diabetic status. While their cardiovascular and renal benefits are well established, euglycemic ketoacidosis (EKA) remains a rare but potentially life-threatening complication that can occur even in non-diabetic individuals.

**Case presentation:**

We report a 58-year-old man with ischemic cardiomyopathy (LVEF 35%) and stage 2 chronic kidney disease who developed nausea, vomiting, and fatigue two weeks after initiating dapagliflozin. Laboratory evaluation revealed high-anion-gap metabolic acidosis (pH 7.21 [reference: 7.35–7.45], HCO_3_^-^ 12 mmol/L [reference: 22–28 mmol/L], anion gap 23 mmol/L [reference: 8–16 mmol/L])with markedly elevated β-hydroxybutyrate (5.4 mmol/L) and normal plasma glucose (108 mg/dL). Diabetes, infection, lactic acidosis, and hepatic dysfunction were excluded.

**Management & outcome:**

The SGLT2 inhibitor was discontinued, and the patient was treated with intravenous saline, insulin infusion, and dextrose. Metabolic parameters normalized within 48 hours, and he was discharged in stable condition. No recurrence was noted at three-month follow-up.

**Conclusion:**

This case highlights that SGLT2 inhibitors can precipitate euglycemic ketoacidosis even in non-diabetic patients. Because normal glucose levels may obscure recognition, clinicians should maintain a high index of suspicion and perform ketone testing in patients on SGLT2 therapy who present with unexplained gastrointestinal or constitutional symptoms.

## Introduction

Euglycemic ketoacidosis (EKA) is a rare but serious metabolic complication characterized by high anion gap acidosis, ketonemia, and relatively normal blood glucose levels (typically <200 mg/dL) (2–4). Traditionally associated with diabetes mellitus, EKA is now increasingly recognized in SGLT2 inhibitor therapy, even in non-diabetic patients (2, 6).

SGLT2 inhibitors, initially developed for glycemic control, are now widely used for heart failure and chronic kidney disease because of their proven cardiovascular and renal benefits. The expanded use in non-diabetic populations has revealed an important safety signal: the risk of EKA extends beyond diabetes (2, 3,7).

Proposed mechanisms include an increased glucagon-to-insulin ratio, enhanced lipolysis, and impaired renal clearance of ketone bodies (3). In non-diabetic patients, preserved insulin secretion prevents hyperglycemia but fails to suppress ketogenesis during stress (3, 5). This paradox delays recognition because clinicians may not suspect ketoacidosis in patients with normal glucose levels (3, 5).

We present a case of severe EKA in a non-diabetic man shortly after initiating dapagliflozin for heart failure.

## Case presentation

A 58-year-old man with a known history of ischemic cardiomyopathy with a left ventricular ejection fraction of 35% and stage 2 chronic kidney disease presented with persistent nausea, repeated non-bilious vomiting, diffuse dull abdominal pain, generalized weakness, fatigue, and loss of appetite for two days. He denied fever, diarrhea, chest pain, worsening breathlessness beyond baseline, altered sensorium, alcohol use, fasting, or recent infection. The patient had been on long-term cardiovascular therapy with aspirin, enalapril, and carvedilol for ischemic cardiomyopathy Three weeks prior to presentation, dapagliflozin 10 mg/day was initiated for heart failure management. The patient had no history of diabetes mellitus.

On evaluation, his body mass index was 26.8 kg/m² (height 172 cm, weight 79 kg). His random blood glucose level was 108 mg/dL, confirming euglycemia. Laboratory evaluation revealed high-anion-gap metabolic acidosis with marked ketonemia in the presence of normal plasma glucose, consistent with euglycemic ketoacidosis (**Table 1**). Arterial blood gas analysis revealed a pH of 7.21, bicarbonate of 12 mmol/L, and pCO_2_ of 26 mmHg, consistent with high anion gap metabolic acidosis; the calculated anion gap was 23 mmol/L (reference range: 8–16 mmol/L). Serum β-hydroxybutyrate was markedly elevated at 5.4 mmol/L (reference: <0.5 mmol/L). The HbA1c level was 5.3%, excluding diabetes, and both insulin and C-peptide levels were within the normal range. Serum lactate levels were normal, ruling out lactic acidosis. Renal function tests showed a creatinine level of 1.6 mg/dL and an estimated GFR of 61 mL/min/1.73 m², consistent with stage 2 chronic kidney disease. The electrolytes were within normal limits.

**Table 1:** Diagnostic laboratory findings at presentation.

Parameter	Result	Reference range
Random Blood Glucose	108 mg/dL	70–140 mg/dL
β-Hydroxybutyrate	5.4 mmol/L	<0.5 mmol/L
Arterial pH	7.21	7.35–7.45
Anion Gap	23 mmol/L	8–16 mmol/L
Serum Creatinine	1.6 mg/dL	0.6–1.3 mg/dL
HbA1c	5.3%	4.0–5.6%
Serum Insulin	6 µIU/mL	3–25 µIU/mL
C-peptide	2.1 ng/mL	0.8–3.5 ng/mL
Urinalysis – Glucose	++	Negative
Urinalysis – Ketones	+++	Negative
Serum bicarbonate (HCO_3_^-^)	12 mmol/L	22–28 mmol/L
pCO_2_	26 mmHg	35–45 mmHg

ABG, arterial blood gas; HCO_3_^-^ = bicarbonate; pCO_2_, partial pressure of carbon dioxide.

Critical metabolic and biochemical parameters confirming the diagnosis of euglycemic ketoacidosis (EKA). Values highlight high−anion−gap metabolic acidosis with markedly elevated ketones in the presence of normal plasma glucose. Routine hematology, and extended urinalysis are provided separately in **Supplementary Table S1**.

Urinalysis revealed glucosuria, attributable to SGLT2 inhibition, and strong ketonuria, consistent with the clinical picture of euglycemic ketoacidosis. Hematology and liver function tests were normal, and chest imaging and echocardiography showed a stable cardiac status without new events.

### Treatment and follow up

Dapagliflozin was discontinued immediately, and the patient was started on intravenous saline to correct dehydration and improve renal clearance of ketones. At presentation, the patient was clinically euvolemic without signs of congestion; therefore, loop diuretics were not required. Mineralocorticoid receptor antagonists were deferred due to underlying chronic kidney disease and concern for hyperkalemia. Heart failure therapy was subsequently optimized after recovery. Continuous insulin infusion was initiated at 0.05 units/kg/hour with 10% dextrose administered concurrently throughout the 48-hour period until acidosis resolved to suppress ketogenesis while maintaining normoglycemia. Blood glucose was monitored hourly, potassium every four hours, and arterial blood gases and ketone levels were regularly reassessed.

Within 48 h, the acidosis resolved with normalization of pH and a marked decrease in ketone levels. The patient became asymptomatic by the third day, tolerated an oral diet (cardiac diet with sodium restriction of < 2 g/day, consistent with heart failure management), and was transitioned from intravenous to subcutaneous insulin, which was subsequently tapered and discontinued. He was discharged on the fourth day in a stable condition, with all metabolic parameters normalized. At follow-up, he remained well with no recurrence, and following recovery, the patient’s heart failure regimen was optimized with initiation of sacubitril-valsartan, while dapagliflozin was permanently discontinued due to euglycemic ketoacidosis.

## Discussion

Euglycemic ketoacidosis represents a diagnostic paradox in contemporary clinical practices. Unlike classic diabetic ketoacidosis, where hyperglycemia serves as an immediate red flag, EKA unfolds insidiously with near-normal glucose levels, often delaying recognition and treatment (3, 5). The increasing use of sodium-glucose cotransporter 2 (SGLT2) inhibitors in non-diabetic populations has broadened the spectrum of patients at risk, underscoring the need for heightened vigilance (2, 3). In the present case, a non-diabetic man developed severe metabolic acidosis with marked ketonemia shortly after the initiation of dapagliflozin for heart failure, highlighting the potential for this complication even in the absence of traditional risk factors.

The pathophysiology of SGLT2 inhibitor-induced EKA is multifactorial (3, 4). By promoting urinary glucose excretion, these agents create a relative carbohydrate-deprived state despite normal dietary intake. This shift in substrate availability increases the glucagon-to-insulin ratio, enhances lipolysis, and drives hepatic ketogenesis (3, 4). In non-diabetic individuals, residual insulin secretion prevents overt hyperglycemia but is insufficient to suppress ketone body formation under physiological stress (3, 5). Moreover, impaired renal clearance of ketones, particularly in patients with chronic kidney disease, further amplifies this risk (3). Therefore, the paradox of normal glucose masking a profound metabolic crisis is a direct consequence of the pharmacological action of SGLT2 inhibition (3, 5).

The differential diagnosis in such cases is challenging. Starvation ketosis was excluded in our patient by the absence of fasting or dietary restrictions (1). Alcoholic ketoacidosis was ruled out based on the negative history and laboratory findings (5). Sepsis, another common precipitant of metabolic acidosis, was excluded based on normal inflammatory markers and sterile cultures. Lactic acidosis was ruled out based on normal lactate levels. The exclusion of these alternative causes strengthens the causal link between dapagliflozin therapy and the development of EKA in this case. Importantly, the patient did not exhibit any of the classic precipitants described in the literature, such as perioperative stress, prolonged fasting, or dehydration (2, 5), suggesting that therapeutic dose dapagliflozin alone may be sufficient to trigger ketosis in susceptible individuals.

Several reports have documented similar presentations in non-diabetic patients. Larroumet et al. described EKA in a non-diabetic individual undergoing therapeutic fasting (7), while Alkatheeri et al. reported a case precipitated by prolonged fasting in a patient on SGLT2 therapy (2). Our case differs in that EKA occurred without any obvious precipitating factor, reinforcing the notion that the risk is not confined to specific stress states but may be intrinsic to the pharmacologic mechanism of SGLT2 inhibition (3, 6). This observation expands the clinical spectrum of EKA and highlights the importance of considering the diagnosis, even in apparently stable patients.

The management of EKA requires a therapeutic approach distinct from that of classic diabetic ketoacidosis (3–5). Immediate discontinuation of the offending agent is essential to prevent severe complications. Fluid resuscitation corrects hypovolemia and improves the renal clearance of ketones, whereas insulin infusion suppresses lipolysis and ketogenesis (3, 4). Concomitant dextrose supplementation is critical to prevent hypoglycemia, given baseline euglycemia (3). Electrolyte monitoring, particularly of potassium, guides supportive therapies (5). In our patient, this regimen led to the rapid resolution of acidosis and complete clinical recovery within 48 hours, consistent with the outcomes reported in other case series (6). The prognosis is excellent when EKA is recognized early, but the risk of recurrence mandates the avoidance of re-exposure to SGLT2 inhibitors (3, 6).

The broader clinical implications of this case report are significant. As SGLT2 inhibitors continue to be prescribed for heart failure and chronic kidney disease, irrespective of diabetic status, clinicians must remain alert to the possibility of EKA in non-diabetic patients (2, 3). The nonspecific nature of the presenting symptoms, such as nausea, vomiting, abdominal pain, and fatigue, can easily be misattributed to cardiac or renal disease, further delaying diagnosis (3, 5). Routine reliance on glucose levels alone is insufficient; ketone testing should be considered in any patient on SGLT2 therapy who presents with unexplained metabolic acidosis or gastrointestinal complaints (3, 6). Education of patients is equally important, as awareness of warning symptoms may prompt earlier medical attention. Finally, clinicians should consider withholding SGLT2 inhibitors during periods of acute illness, surgery, or reduced oral intake when the risk of ketosis is heightened (2, 3).

In summary, this case adds to the growing body of evidence that dapagliflozin and other SGLT2 inhibitors can precipitate euglycemic DKA, even in non-diabetic patients, sometimes in the absence of traditional precipitants (2, 6). The paradox of normal glucose concealing a life-threatening metabolic derangement underscores the need for clinical vigilance, timely ketone testing, and prompt intervention (3, 5). Recognition of this complication is essential to ensure safe prescribing practices, as the therapeutic use of SGLT2 inhibitors continues to expand beyond diabetes (2, 3).

## Conclusion

SGLT2 inhibitors provide substantial benefits in heart failure and chronic kidney disease but carry a rare risk of euglycemic ketoacidosis, even in non-diabetic patients. Normal glucose levels (<200 mg/dL) may mask early recognition, underscoring the need for ketone testing in patients on SGLT2 inhibitors who present with unexplained gastrointestinal or constitutional symptoms. Clinicians should exercise caution, educate patients about warning signs, and withhold therapy during acute illnesses or stress.

## Data Availability

The original contributions presented in the study are included in the article/**Supplementary Material**. Further inquiries can be directed to the corresponding author.

## Supplementary material

Supplementary Table 1 Baseline routine laboratory investigations at presentation. CBC = complete blood count; TLC = total leukocyte count; ESR = erythrocyte sedimentation rate; HPF = high-power field.
