# Supplementary material for: Case Report: Euglycemic ketoacidosis in a non-diabetic patient: a rare adverse effect of SGLT2 inhibitor therapy
Source: Front Endocrinol (Lausanne). 2026 Jan 16;16:1746210. doi: 10.3389/fendo.2025.1746210 (PMC12855120; doi:10.3389/fendo.2025.1746210)
Supplement: Supplementary Table 1 — Baseline routine laboratory investigations at presentation. CBC = complete blood count; TLC = total leukocyte count; ESR = erythrocyte sedimentation rate; HPF = high-power field. [file Table1.docx]

**Supplementary Table S1**

**Baseline Routine Laboratory Investigations at Presentation**

| **Parameter** | **Result** | **Reference Range** |
| --- | --- | --- |
| **Complete Blood Count (CBC)** |  |  |
| Haemoglobin | 14.1 g/dL | 13.0 – 17.0 g/dL |
| Haematocrit | 43.2 % | 40 – 50 % |
| TLC | 8,900 /mm³ | 4,000 – 11,000 /mm³ |
| Neutrophils | 68 % | 40 – 75 % |
| Lymphocytes | 25 % | 20 – 45 % |
| Monocytes | 5 % | 2 – 10 % |
| Eosinophils | 2 % | <6 % |
| Platelets | 2.8 × 10⁵ /µL | 1.5 – 4.0 × 10⁵ /µL |
| ESR | 14 mm/hr | <20 mm/hr |
| **Urinalysis (Routine & Microscopy)** |  |  |
| Colour | Pale yellow | Pale yellow amber |
| Appearance | Clear | Clear |
| Specific Gravity | 1.030 | 1.005 – 1.030 |
| pH | 5.0 | 4.5 – 8.0 |
| Protein | Trace | Negative |
| Glucose | ++ | Negative |
| Ketones | +++ | Negative |
| Blood | Negative | Negative |
| Leukocyte Esterase | Negative | Negative |
| Microscopy: Pus cells | 0–2 /HPF | 0–5 /HPF |
| Microscopy: RBCs | 0–1 /HPF | 0–2 /HPF |
| Bacteria | Nil | Nil |

CBC = complete blood count; TLC = total leukocyte count; ESR = erythrocyte sedimentation rate; HPF = high-power field**.**
